# Perceptions of Multiple Perpetrator Rape in the Courtroom

**DOI:** 10.3390/bs15070844

**Published:** 2025-06-23

**Authors:** Kelly C. Burke, Jonathan M. Golding, Jeffrey Neuschatz, Libbi Geoghagan

**Affiliations:** 1 Department of Psychology, University of Texas at El Paso, El Paso, TX 79968, USA; 2 Department of Psychology, University of Kentucky, Lexington, KY 40506, USA; 3 Applied Psychological Sciences Department, Fielding Graduate University, Santa Barbara, CA 93105, USA; 4 Department of Psychology, University of Alabama in Huntsville, Huntsville, AL 35899, USA

**Keywords:** victimization, gang rape, multiple perpetrator rape, court, legal decision making, juror decision making

## Abstract

Rape is typically committed as a one-on-one crime. However, a relatively high number of rapes (2–27%) involve a single victim and multiple perpetrators. These cases are often referred to as “gang” rapes but are also termed Multiple Perpetrator Rape (MPR). Despite these data, there is a scarce amount of legal decision-making research on this issue. This study investigated legal decision making in an acquaintance rape case involving multiple perpetrators. This study was a 2(Defendant Number: one vs. three) × 2(Victim Intoxication: intoxicated vs. sober) × 2(Participant Gender: women vs. men) between-participants design. Online community members (*N* = 171) were randomly assigned to read a trial summary involving one of four conditions. The primary results showed that, when the case involved multiple (vs. one) perpetrators, mock jurors were more likely to vote guilty, perceived the victim to be more helpless, and reported less sympathy for the defendant and lower defendant credibility. Cognitive networks showed that jurors in the MPR condition emphasized the number of perpetrators as a primary reason for voting guilty. Finally, there was evidence of a serial indirect effect involving victim helplessness and defendant blame that explained the relation between the number of defendants and verdicts, as well as parallel indirect effects of defendant credibility, sympathy, and anger, and victim helplessness on verdicts. Implications for prosecuting MPR cases are discussed.

## 1. Introduction

Despite national efforts in the US to prevent adult rape (e.g., Rape Prevention and Education Program, 2024), this crime continues to be prevalent. For example, in 2022, there were 133,294 reported rape cases (including attempted rapes) (Statista, 2022). Yet, rape is underreported, and the most recent estimates suggest that 2.9 million women were victims^1^ of rape in the US in 2016–2017 (Basile et al., 2022). Unfortunately, despite its prevalence, few rape cases are prosecuted—an estimated 13 cases out of every 1000 instances of rape are referred for prosecution (RAINN, 2025b; see also Walinchus et al., 2025)—and an even smaller percentage (7 cases out of every 1000) results in felony convictions (RAINN, 2025b; Walinchus et al., 2025).

Rape impacts the country in negative ways at all levels of society. At the micro (i.e., individual) level, victims experience significant difficulties including self-blame, depression, post-traumatic stress disorder, the fear of being stigmatized or not believed, among a host of other devastating consequences (RAINN, 2025a). The trauma they experience can impair their daily lives as well as their relationships with others (O’Callaghan et al., 2021). At the meso (i.e., community) level, when rapists are not prosecuted or brought to justice, this can result in the victimization of others (Hanson et al., 2022; Przybylski, 2015) and a loss of trust in the legal system among victims, families, and others who perceive that justice has not been served (Wieberneit et al., 2024). Rape also strains financial resources and mental health (e.g., vicarious trauma) among those pursuing justice against perpetrators of rape as well as those providing services to victims (e.g., law enforcement, social services, healthcare; Crivatu et al., 2023; Peterson et al., 2017). At the macro (i.e., societal) level, norms that tolerate sexual exploitation and violence can perpetuate negative stereotypes and skepticism over rape allegations and contribute to a “rape culture” (Canan & Levand, 2019; Johnson & Johnson, 2021).

The purpose of the present study was to investigate legal decision making involving an acquaintance rape trial in US criminal court when the rape involved multiple perpetrators, otherwise known as “gang rape.” Although any individual may be the victim of rape (e.g., men, non-binary persons, children), we focus on rape involving adult men as perpetrator(s) and an adult woman as the victim, because adult women are among those at the highest risk of being victimized in the US (90% of adult rape victims are female; RAINN, 2025a). Moreover, gender- and sex-based stereotypes as well as attitudes that foster patriarchy, misogyny, gender inequality, and heteronormativity foster the perpetration of rape against women (Ullman, 2013). Indeed, as Woodhams and Horvath (2013) note, “…the extent to which women are treated as sexual objects to be passed around, humiliated and denigrated in the pursuit of male bonding and achieving enhanced masculine status is far more apparent in MPR [multiple perpetrator rape] than in LPR [lone perpetrator rape]” (p. 3; see also Kelly, 2013).

There are several important points to note about acquaintance rape. First, acquaintance rape is the most common type of adult rape in the US (Basile et al., 2022; Golding et al., 2023). Second, like other types of rape, acquaintance rape is typically defined by three critical components in US federal and state law (e.g., FBI, 2013; Illinois Criminal Code §§ 5/11-1.20, n.d.; Kentucky Penal Code §§ 510.040-510.060, n.d.). These include the following: (a) contact between a victim’s genital, anal, or oral areas and the perpetrator’s genitals, hand, or an object used by the perpetrator; (b) the use of physical force or the threat of force to complete the act; and (c) the absence of victim consent or the inability for a victim to give consent (i.e., nonconsensual). Third, although most rape cases hinge on the third component of consent, this component is especially important in acquaintance rape cases. This is because the victim and defendant typically acknowledge that sexual intercourse occurred; however, the victim argues that there was no consent, whereas the defendant argues that consent was provided. Thus, unlike stranger rape cases, where the identification of the perpetrator is the crucial issue at hand, in acquaintance rape, the crucial issue is establishing the nonconsensual nature of the rape. As a result, decision making in these cases often boils down to a “she-said-he-said” battle (Golding et al., 2023). Finally, in cases of acquaintance rape, victims may feel even more helpless and offer less resistance compared to cases involving a stranger (Schafran, 2005).

Although rape is typically committed as a one-on-one crime (RAINN, 2024), rape can occur with multiple perpetrators. Horvath and Kelly (2009) proposed a term to describe such rape: Multiple Perpetrator Rape (MPR). Regarding prevalence, on a global scale, the prevalence of MPR cases varies considerably.^2^ For instance, approximately 32% of South African men who had committed rape had done so with other perpetrators (Jewkes et al., 2015). In Cambodia, MPR was more prevalent than single-perpetrator rape (5.2% vs. 3.1%, respectively; Jewkes et al., 2013). In the US (the focus of the present study), 6% of rapes can be classified as MPR (RAINN, 2024). Internationally, da Silva and Woodhams (2019) noted that approximately 9–33% of rapes were MPR. This is consistent with Woodhams and Horvath (2013), who estimate that MPR ranges from 2 to 27% of all rapes (Horvath & Kelly, 2009; Vetten & Haffejee, 2005). MPR cases may not occur as often as single-perpetrator rapes, but, because they are so shocking, they often find their way into the media (e.g., the Jyoti Singh Pandey (“Nirbhaya”) case in Delhi, India, which sparked widescale national and international protest and media attention, Arya, 2022; the alleged gang rape of a high school student by football players at San Diego State University was “one of the most watched criminal investigations in the country,” Figueroa et al., 2022).

The issue of MPR has received relatively little attention in the victimization literature; however, a volume edited by Horvath and Woodhams (2013) offers critical insight into this type of crime. Several points are worth noting (see Kelly, 2013; Woodhams & Horvath, 2013). First, MPR involves disrespect and humiliation, given that the rape is witnessed by at least one other person (Kelly, 2013). Second, MPR, by definition, is a form of coercion (Kelly, 2013). Third, compared to a single-perpetrator rape, MPR often involves the greater use of physical violence, more severe sexual assault, and the use of a weapon (Kelly, 2013; Woodhams & Horvath, 2013).

The limited research that exists on legal decision making in MPR cases suggests that people endorse similar rape myths (i.e., stereotypes about what defines a “real” rape and stereotypical characteristics of the victim and offender such as only “real” victims resist an attack, Stuart et al., 2019) as they do with single-perpetrator cases (Lees, 2002). In addition, in an archival study, Horvath and Gray (2013) examined the kinds of MPR that reach court and the verdicts in these cases. Of the 33 MPR cases that they examined from England and Wales in 2011, 25 of the cases involved victims 13 years old and above. Of these 25 cases, about half (12) involved the victim knowing their attackers, in 7 of the cases, the perpetrators were strangers, and in 6 cases the relationship between victim and perpetrator was not known. Moreover, in the 12 known-perpetrator cases, most of the perpetrators were found guilty (92%), whereas this percentage was only 57% when the perpetrators were strangers, and only 33% when the relationship between the victim and perpetrators was not known.

Horvath and Gray (2013) offered an important first look at MPR and legal decision making. Most important in this regard, they showed that the majority of MPR cases, particularly those involving acquaintance rape, lead to guilty verdicts. However, because this study was based on archival data and only presents verdict data without a comparison group (e.g., rape cases involving a lone perpetrator), it is difficult to fully understand the nature of legal decision making when MPR cases reach court. For example, no data were presented that explored jurors’ perceptions of the case (e.g., victim/defendant credibility, reasons for their verdict).

The dearth of research involving MPR and legal decision making is in stark contrast to the vast literature investigating acquaintance rape and legal decision making involving a lone perpetrator. This literature was recently reviewed by Golding et al. (2023) and showed the influence of extra-legal factors (i.e., not within the scope of the law) in these cases. For instance, participant gender has a robust impact on the outcome of these cases. As one example, in a study involving acquaintance rape, Belyea and Blais (2023) found that women were more pro-victim (e.g., rendered more guilty verdicts) than were men.

Another extra-legal factor that has garnered a great deal of attention concerns the impact of rape victim intoxication. We should note that rape victims are often intoxicated due to alcohol at the time of a rape (Richer et al., 2017). A victim’s intoxication at the time of a rape may lessen the likelihood that a prosecutor brings a case to court because of concerns over the victim’s credibility or jurors’ perceptions of the victim’s credibility (Fansher & Welsh, 2023; Spohn & Tellis, 2019). Research has generally shown that the concerns prosecutors hold about victim intoxication in court are warranted. For example, Schuller and Wall (1998; see also Martin & Monds, 2024) found that an intoxicated victim was perceived as less credible compared to a victim who was sober. In addition, Wall and Schuller (2000) found that mock jurors perceived the victim to be less credible and more responsible when the victim was moderately or extremely intoxicated compared to when the victim was sober.

### The Present Study

The present study extended prior legal decision-making research investigating rape cases involving a lone defendant to cases involving MPR. We used a 2(Defendant Number: one vs. three) × 2(Victim Intoxication: intoxicated vs. sober) × 2(Participant Gender: women vs. men) between-participants design. Online community members received one of four trial summaries, each describing the rape of a female victim by a man whom the victim met at a party (i.e., acquaintance rape). Participants provided a verdict and reason(s) for their verdict. They also rated the victim and defendant on various measures (e.g., credibility, responsibility). Based on prior research, we had four hypotheses:

**Hypothesis** **1.** *Main Effect of Defendant Number.*

Given that victims of rape often report feeling helpless (Schafran, 2005), we theorized that the presence of multiple perpetrators may imbue an added degree of perceived helplessness and vulnerability on the part of the victim, prompting jurors to render harsher judgments of the defendants. Therefore, compared to an acquaintance rape involving one defendant, we hypothesized that *a case involving three defendants would lead to higher pro-victim judgments* (e.g., more guilty verdicts, higher perceived victim helplessness) and lower defendant judgments (e.g., lower defendant credibility).

**Hypothesis** **2.** *Main Effect of Victim Intoxication.*

We predicted that *when a victim was intoxicated, participants would render fewer guilty verdicts* than if the victim was sober at the time of the incident. This was based on prior research showing that mock jurors perceive intoxicated (vs. sober) victims to be less credible and are less likely to convict defendants in these cases (e.g., Martin & Monds, 2024; Schuller & Wall, 1998; Wall & Schuller, 2000).

**Hypothesis** **3.** *Main Effect of Participant Gender.*

As found in most legal decision-making research involving rape cases (see Golding et al., 2023 for review), we predicted that *women would render more pro-victim judgments* (e.g., guilty verdicts, higher ratings of victim credibility) than would men.

**Hypothesis** **4.** *Reason(s) for Verdict Qualitative Data.*

We predicted that the open-ended reasons mock jurors gave for why they voted guilty would differ as a function of the number of defendants involved. *In the three-defendant condition, we expected that the primary reasons participants mentioned for voting guilty would emphasize factors specific to MPR such as the presence of multiple perpetrators and the victim’s helplessness* (Woodhams & Horvath, 2013). *In contrast, in the one-defendant condition, we expected that participants would note that a rape occurred but would not explicitly refer to the nature of there being a single, lone perpetrator as a reason for voting guilty*.

The open-ended reasons participants gave for their verdict allowed us to generate a specific cognitive representation of the case (i.e., cognitive network). This network was created using a combination of natural language processing techniques and the psychometrically established Pathfinder data scaling algorithm (Schvaneveldt, 1990). The creation of these networks has been used effectively in other legal decision-making studies involving victimization (e.g., Levi et al., 2022) and provides additional insight into jurors’ decision making that is not offered by inferential statistics.^3^ For instance, Levi et al. (2022) created cognitive networks scaled by Pathfinder to examine the effect of attorney gender in a rape trial involving a female victim and male defendant. These analyses revealed gender-based stereotypes in jurors’ reasoning for why a male versus female prosecutor or defense attorney would be assigned to the case. Specifically, jurors presumed that female attorneys (defense and prosecution) would be more sensitive and sympathetic to a jury in a rape case. In contrast, they believed a male attorney (defense or prosecution) would be more competent, less biased, and more authoritative.

**Exploratory** **Hypotheses.** *Indirect Effects.*

Given the limited research on MPR, we were interested in exploring which perceptions of the victim and/or defendant (credibility, blame, sympathy, anger, helplessness, consent) best predicted the relation between the number of defendants and the dependent variable of verdict. Prior research has found indirect effects through victim credibility, defendant credibility, and sympathy toward the victim in adult rape trials (e.g., Lynch et al., 2013). As such, we predicted that the strongest parallel indirect effects would consist of these three perceptions such that *participants who received a trial in which there were three (vs. one) defendants would perceive the victim to be more credible, have more sympathy toward the victim, perceive the defendant to be less credible, and in turn be more likely to vote guilty*.

In addition, we predicted that the effect of the number of defendants (one vs. three) would indirectly influence participant verdicts through perceptions of the victim’s helplessness and defendant blame. *Specifically, we expected that participants in the three-defendant condition would perceive the victim to be more helpless, which would be associated with perceiving the defendant to be more blameworthy, and in turn being more likely to vote guilty (i.e., serial indirect effects).* This prediction builds upon Hypothesis 1 suggesting that victims of MPR may feel especially helpless and that such helplessness may translate into higher perceived blame on the part of the defendants.

## 2. Method

### 2.1. Participants

Participants included 228 US community members recruited from Mechanical Turk. Fifty-seven participants (23.3%) were excluded from analyses for several reasons. First, consistent with US laws pertaining to juror eligibility, three participants were excluded for not being US citizens. Second, because the number of participants identifying as “Other” (one participant) or “Transgender” (one participant) was too low for meaningful analyses on these categories, these participants were excluded. Third, 18 participants were excluded for not completing the experiment. Fourth, 21 participants were excluded based on recent concerns about the validity of Mechanical Turk participants (e.g., Webb & Tangney, 2022). This led to five participants being excluded because of our criteria for BOTs (e.g., single-word responses to our open-ended item asking about reason(s) for rendering a verdict), five excluded for listing the same worker ID, and eleven excluded for having the same IP address. Fifth, 10 participants were excluded for answering the manipulation checks about the number of defendants and the victim’s intoxication incorrectly. Sixth, one participant was excluded for answering more than one of the six attention-check questions incorrectly. Finally, two participants from the three-defendant condition were excluded because they did not have the same verdict for each of the three defendants, thus precluding meaningful analyses. The final sample included 171 participants.

We should note that the following protocols were also employed to ensure a valid sample: (a) the use of a factorial design (described in the Design Subsection), in which participants were randomly distributed across conditions to ensure that they were unaware of their experimental condition; (b) six attention-check questions were asked throughout the trial to ensure that participants were reading the text and paying attention; (c) participants were unable to go back to re-read text or answer questions, making the concern with click counts (see Buchanan & Scofield, 2018) not applicable; (d) all participants were run using Cloud Research Approved and Blocked Groups, determined by Hauser et al. (2023) to be valid predictors of data quality in social science surveys; (e) the use of Cloud Research allowed us to block Mechanical Turk participants from accessing similar surveys using a mock-trial methodology; and (f) Cloud Research allowed us to indicate an HIT Approval Rate of 95–100% (Hauser et al., 2023) to ensure high-quality data.

Prior research examining perceptions of adult rape and the influence of participant gender (Lynch et al., 2013; Schuller et al., 2013; Schuller & Wall, 1998; Wall & Schuller, 2000) and victim intoxication (Jenkins & Schuller, 2007; Lynch et al., 2013; Martin & Monds, 2024; Schuller & Wall, 1998) found small-to-medium effects. Based on these effects, a power analysis using the pwrss package in R (Bulus, 2023) indicated that 133 participants would produce sufficient power (0.80) to detect small-to-medium (f^2^ = 0.06) main effects in the hierarchical linear regression. For dichotomous verdict, an odds ratio of 2.44 was estimated based on this prior research. We estimated 0.4 probability of the null given that we expected participants in the single-perpetrator and intoxicated conditions as well as men to vote guilty 40% of the time, *R*^2^ of other Xs = 0 because we did not expect the experimental manipulations to be correlated with other variables in the regression, and × parm = 0.5 because we expected an even split between conditions. This resulted in a minimum of 164 participants to detect the main effects with sufficient power (0.80).

Of the final sample, on average, participants were 43.67 years old (*SD* = 13.14), ranging from 21 to 78 years, with 46.8% of participants identifying as female and 53.2% identifying as male. Participants identified as White/Caucasian (74.3%), Black/African American (11.7%), Asian or Pacific Islander (5.3%), Hispanic/Latinx (5.3%), multi-race and ethnicity (2.3%), and other (1.1%). A relatively low number of participants indicated that they had previous jury service (15.8%).

### 2.2. Design

The experiment was a 2(Defendant Number: one vs. three) × 2(Victim Intoxication: intoxicated vs. sober) × 2(Participant Gender: women vs. men) between-participants design. The primary dependent measures included a verdict for the defendant and various measures of the victim’s and defendant’s credibility and blame.

### 2.3. Materials

Trial Summary: Participants were presented with a written summary of a fictitious criminal trial of rape in the first degree. Development of the trial summaries was based on the field’s guidelines and norms for implementing a mock trial methodology (for reviews and discussion of this method, see, e.g., Bornstein et al., 2017; Bray & Kerr, 1979; Burke et al., 2025; Diamond, 1997; Golding et al., 2020; Ross, 2024).^4^ All witnesses received direct and cross-examination. The one-defendant condition had one male defendant, and the three-defendant condition included three male defendants (see online Supplementary Materials for full study materials). 

In the one-defendant condition, the prosecution’s case included a female victim testifying that she attended a party at the home of the defendant. At the party, she was approached by the defendant, and they started talking. The victim stated that she either drank beer or water at the party. At the end of the night, the victim stated that the defendant offered to give her a tour of the house. They ultimately ended up in the defendant’s bedroom. She stated that the defendant forced her into his bedroom, shut and locked the door, and proceeded to initiate sexual intercourse with her. The victim stated that she did not want to have sexual intercourse, nor did she consent to have sexual intercourse. The three-defendant condition included the above testimony and added testimony from the victim that the defendant contacted two other men via text. These other men entered the room and forced her to have sexual intercourse with them. In both conditions, another witness, a police officer, testified for the prosecution. The officer testified that the victim appeared disheveled upon his arrival and that she had bruises on her arms but also acknowledged that he did not know exactly why she was in this state.

The defense’s case began with a witness who was at the party. The witness stated that they did not observe the defendant do anything aggressive or sexual toward the victim. The defendant also testified that he had not been drinking alcohol the night of the party. He admitted to having consensual sex with the victim but clarified that she initiated and consented to the encounter, after she suggested that they both go upstairs to his bedroom. The defendant emphasized that he did not force her to have sex with him. In the three-defendant condition, the initial defendant stated that he did text two friends to come over to his home, but that the victim consented to have the other men join them. The other two defendants also testified that the victim was willing to have them join. Of note, although the number of witnesses testifying for the defense differed across conditions (i.e., one versus three defendants), the additional defendants in the three-defendant condition would likely work against our hypotheses, given prior research showing that the more witnesses who testify for the defense, the less likely jurors are to convict (e.g., Werner et al., 1985).

After all witnesses testified, participants were presented with instructions from the judge and closing arguments.

#### Trial Questionnaire

Participants answered a series of questions about the trial after reading one of the trial summaries. They rated the guilt of the defendant (1 = *Not at all* to 7 = *Completely*) and then provided a dichotomous verdict (*Not guilty* or *Guilty*). Next, participants provided an open-ended response explaining the reason for their chosen verdict (“Why did you choose your verdict?”).

Following this, participants responded to questions about the victim and defendant. Specifically, participants rated the intoxication level, credibility, honesty, and believability of the victim and defendant(s), how responsible and blameworthy the victim and defendant(s) were for the incident, and sympathy and anger toward the victim and defendant(s). An example item was, “How sympathetic are you toward the victim?” Responses were made on a scale ranging from 1 (*Not at all*) to 7 (*Completely*). Other questions concerning only the victim (using the same scale) included rating her physical helplessness, ability to communicate, ability to consent, and memory accuracy for the incident. Other questions for the defendant(s) (using the same scale) included the amount of force used on the victim and the defendant’s perception of the victim’s vulnerability.

### 2.4. Procedure

This study was conducted according to the guidelines of the Declaration of Helsinki and approved by the Institutional Review Board of the University of Kentucky. All American Psychological Association (APA) ethical guidelines were followed (American Psychological Association, 2017).

Participants completed this study online through the Qualtrics survey platform. First, participants read the informed consent form and indicated yes/no whether they agreed to participate in this study. Next, participants were instructed to read the trial summary and answer questions as they were displayed. During the presentation of the trial, manipulation-check questions asked how many men were on trial for raping the victim and whether the victim drank beer or water at the party. There were also six attention-check questions (e.g., “What did Officer Williams say about Ms. Spellman’s clothes the night of the crime?”) throughout the summary to ensure that participants were reading carefully and understanding the details of the case. When a participant answered any of these questions incorrectly, they were advised to read the summary carefully. After the trial summary, participants gave their judgments of the trial. Last, they provided the following demographic information: citizenship (“Are you a citizen of the United States? yes/no”); sex (“Are you: male, female, transgender, other”); age (“What is your age?”); race/ethnicity (“What is your race? Caucasian, African American, Native American or Alaska Native, Asian or Pacific Islander, Hispanic or Latinx, Other (open-ended)”); and prior jury service (“Have you ever served on a jury? yes/no”; “If yes to the previous question, how many times? For each case you served as a juror, what was the crime charged and what was the verdict?”). Once participants completed this study, they were debriefed and given a copy of the consent form.

### 2.5. Data Analytic Plan

Several steps were followed to analyze the data. First, for the three-defendant condition, the three defendant ratings for each dependent variable were combined into a single score. Second, four composite scores were created: (a) a victim credibility scale comprised the average of four rating variables: victim credibility, believability, honesty, and accuracy (Cronbach’s α = 0.94); (b) a victim responsibility/blame scale comprised the average of two rating variables: victim responsibility and blame (Cronbach’s α = 0.92); (c) a defendant credibility scale comprised the average of three rating variables: defendant credibility, believability, and honesty (Cronbach’s α = 0.99); and (d) a defendant responsibility/blame scale comprised the average of two rating variables: defendant responsibility and blame (Cronbach’s α = 0.96). Prior work used similar items to assess defendant and victim responsibility (e.g., Saulnier et al., 2019; Wiley & Bottoms, 2013) and credibility (e.g., Regan & Baker, 1998; Ruva et al., 2023; Tenney et al., 2007). Next, the verdict and decision data were analyzed using regression. A logistic regression was used for verdict due to the dichotomous nature of this variable. The rating questions were analyzed using linear regression. For all regressions, Step 1 included Participant Gender (0 = woman, 1 = man). In Step 2, Defendant Number (0 = one, 1 = three) and Victim Intoxication (0 = intoxicated, 1 = sober) were entered.

To construct cognitive networks from the reasons that participants gave for rendering a guilty verdict, we first preprocessed the sets of open-ended responses. Each response was tokenized into individual terms, and common stop words (e.g., “the,” “is,” “at,” “which”) were removed. We then lemmatized the terms to reduce words to their base forms (e.g., “walking” to “walk”) and applied part-of-speech tagging to identify grammatical categories (e.g., “noun,” “verb”). We also removed punctuation and excluded terms longer than 15 characters to reduce noise.

Next, we constructed a term-by-response matrix, where each row represents a unique term, and each column corresponds to a participant’s response. The matrix entries reflect the Term Frequency–Inverse Document Frequency (TF-IDF) score of each term within each response. TF-IDF is a widely used statistical method in natural language processing and information retrieval that is used to identify contextually important keywords. To create a rank ordering of the importance of the terms, we computed the average TF-IDF score for each term across all responses.

From this ranked list, we selected any keywords that were at the median level of the total distribution or above, based on both their rank and our interpretive relevance.^5^ We then computed the cosine distance between all pairs of these selected terms based on their TF-IDF score vectors across responses, which resulted in a term-by-term distance matrix. This distance matrix served as the input to the Pathfinder network scaling algorithm (Schvaneveldt, 1990). Two parameters, *r* and *q*, govern how Pathfinder prunes connections in the network. We opted for the default parameters, *r* = *∞* and *q* = n − 1 (where n = number of nodes), which yield a conservative pruning strategy that only retains direct links when no stronger indirect alternative exists. MATLAB (2024) was used to create the cognitive networks using Schvaneveldt’s (1990) original code.

Finally, to test the indirect effects of victim and defendant perceptions, we used structural equation modeling via the lavaan package (Rosseel, 2012) in R version 2024.12.1+563 (R Core Team, 2024).^6^ This enabled us to model covariances among variables given that variables were highly correlated with each other and raised concerns over multicollinearity.

## 3. Results

Participants’ ratings of the victim’s and defendants’ intoxication levels supported the descriptions in the trial summaries. When the victim was described as “wasted,” the mean rating was 6.57 (*SD* = 0.88), and when described as sober, the mean rating was 1.19 (*SD* = 0.84) (*F*(1, 169) = 1658.90, *p* < 0.001). The defendant(s), who were always described as sober, had an overall mean rating of 1.67 (*SD* = 1.65). Regarding all other dependent variables, see Table 1 for means and standard deviations.

**Hypothesis** **1.** *Main Effect of Defendant Number.*

As predicted, the logistic regression for the verdict yielded a main effect of the number of defendants (*B* = 1.05, SE = 0.32, *Wald’s χ*^2^ = 10.84, *OR* = 2.86, *p* < 0.001) (see Table 2 for full regression results). The presence of three defendants increased the odds of a guilty verdict 2.86 times relative to when there was only one defendant. In addition, three (vs. one) defendants led participants to perceive the victim to be more helpless (*B* = 0.87, SE = 0.26, β = 0.23, *t*(167) = 3.32, *p* = 0.001) and the defendants less credible (*B* = −0.95, SE = 0.26, β = −0.27, *t*(166) = −3.70, *p* < 0.001), as well as to report less sympathy for the defendants (*B* = −0.81, SE = 0.26, β = −0.23, *t*(167) = −3.10, *p* < 0.001).

**Hypothesis** **2.** *Main Effect of Victim Intoxication.*

There was no support for the prediction that participants would be more likely to convict the defendant when the victim was sober versus intoxicated (55% of participants in the sober condition voted guilty, 46% of participants in the intoxicated condition voted guilty). However, participants did perceive the intoxicated victim to be more helpless (*B* = −1.50, SE = 0.26, β = −0.39, *t*(167) = −5.69, *p* < 0.001), less able to communicate (*B* = 1.85, SE = 0.26, β = 0.49, *t*(167) = 7.25, *p* < 0.001), and less able to consent (*B* = 2.04, SE = 0.29, β = 0.48, *t*(166) = 6.93, *p* < 0.001). Participants also reported that the defendant perceived the victim to be more vulnerable when she was intoxicated versus sober (*B* = −1.20, SE = 0.30, β = −0.30, *t*(167) = −4.03, *p* < 0.001).

**Hypothesis** **3.** *Main Effect of Participant Gender.*

Consistent with the prediction that women would show more pro-victim perceptions of the case than men, women reported higher victim credibility (*B* = −0.50, SE = 0.25, β = −0.16, *t*(169) = −2.06, *p* = 0.04), less anger toward the victim (*B* = 0.57, SE = 0.24, β = 0.18, *t*(169) = 2.34, *p* = 0.02), lower victim responsibility/blame (*B* = 0.64, SE = 0.26, β = 0.18, *t*(169) = 2.42, *p* = 0.02), and more anger toward the defendant (*B* = −0.71, SE = 0.34, β = −0.16, *t*(169) = −2.12, *p* = 0.04).

**Hypothesis** **4.** *Reason(s) for Verdict Qualitative Data.*

**Figure 1:**
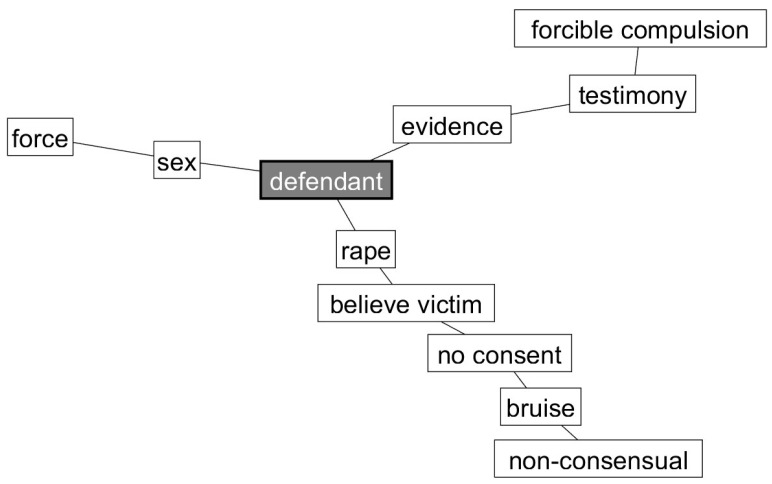
Pathfinder networks of a guilty verdict in the one-defendant condition.

**Figure 2:**
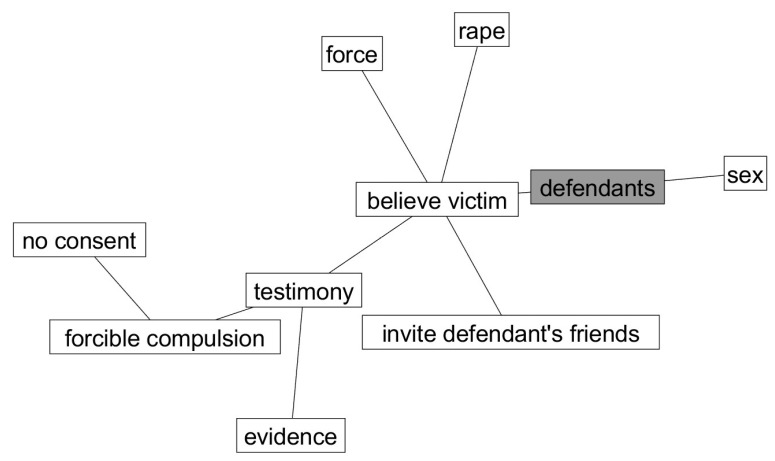
Pathfinder networks of a guilty verdict in the three-defendant condition.

This hypothesis received some support. As predicted, the cognitive network for participants who rendered a guilty verdict in the one-defendant condition (see Figure 1) did mention that a rape had occurred. Regarding their mention of the defendant, it was the primary reason for a guilty verdict, but participants did not specify that “one” defendant had committed a crime. Regarding the three-defendant condition (see Figure 2), the prediction that participants would note the victim’s helplessness as a reason was not supported. However, as predicted, participants noted multiple perpetrators (i.e., “defendants”) as the primary reason for their verdicts and emphasized the presence of multiple perpetrators (i.e., “invite defendant’s friends”) as an important reason for voting guilty.

**Exploratory** **Analyses.** *Indirect Effects.*

We explored whether the direct effect of the number of defendants on the verdict was predicted by indirect effects through one or more third variables. We tested a model with perceptions of the victim and defendant as parallel indirect effects (*m*) between the defendant number (*x*) and verdict (*y*) to better understand which perceptions were strongest in predicting the relationship between MPR and the verdict decision. These perceptions included victim/defendant credibility, victim/defendant blame, victim/defendant sympathy, victim/defendant anger, victim helplessness, and victim consent. Results revealed indirect effects of defendant credibility, defendant sympathy, defendant anger, and victim helplessness that accounted for the relationship between the number of defendants and verdicts. Specifically, relative to the one-defendant condition, participants in the three-defendant condition perceived the defendant to be less credible (*B* = −1.05, SE = 0.27, *p* < 0.001), had less sympathy for the defendant (*B* = −0.89, SE = 0.29, *p* = 0.002), were angrier at the defendant (*B* = 0.75, SE = 0.36, *p* = 0.04), and perceived the victim to be more helpless (*B* = 0.75, SE = 0.30, *p* = 0.01). Each of these were associated with an increased likelihood of voting guilty: lower defendant credibility (*B* = −0.50, SE = 0.03, *p* < 0.001), lower defendant sympathy (*B* = −0.42, SE = 0.03, *p* < 0.001), more anger at the defendant (*B* = 0.32, SE = 0.02, *p* < 0.001), and higher perceived victim helplessness (*B* = 0.31, SE = 0.03, *p* < 0.001) predicted guilty verdicts (see Table 3 for all indirect effects).

**Figure 3:**
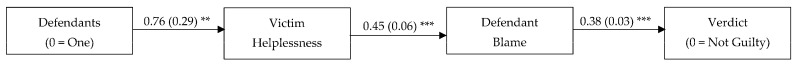
Serial indirect effects of victim helplessness and defendant blame on the relation between defendant number and verdict note. Coefficients reflect unstandardized betas with standard errors in parentheses. ** *p* < 0.01, and *** *p* < 0.001.

In addition, we tested a model in which victim helplessness and defendant blame were serial indirect effects between the number of defendants and the verdict. As predicted, results showed evidence of a serial indirect effect (*B* = 0.13, SE = 0.05, *p* = 0.01). Specifically, compared to the one-defendant condition, participants in the three-defendant condition perceived the victim to be more helpless (*B* = 0.76, SE = 0.29, *p* = 0.01), which was associated with higher perceptions of defendant responsibility/blame (*B* = 0.45, SE = 0.06, *p* < 0.001), and in turn a greater likelihood of voting guilty (*B* = 0.38, SE = 0.03, *p* < 0.001) (see Figure 3).

## 4. Discussion

The present study investigated US legal decision making in an adult rape case involving multiple male perpetrators through the opinions of survey participants. Our primary findings were that, compared to a rape case with a single perpetrator, a multiple perpetrator rape case led to the following: (a) an increase in guilty verdicts; (b) higher ratings of victim helplessness; (c) lower ratings of defendant credibility and sympathy; (d) reasons for a guilty verdict that emphasized the presence of other perpetrators; (e) evidence of indirect effects of defendant credibility, defendant sympathy, defendant anger, and victim helplessness; and (f) a serial indirect effect involving victim helplessness and defendant blame. With respect to the effect of victim intoxication, contrary to predictions, participants were not more lenient toward the defendant when the victim was described as intoxicated compared to sober. Yet, we did find evidence of gender effects similar to prior work (e.g., Belyea & Blais, 2023) indicating that women made more pro-victim judgments than did men.

Our findings are consistent with Horvath and Gray’s (2013) archival research showing that MPR cases led to a high number of guilty verdicts. We extended these earlier findings by using an experimental approach that allowed direct, causal comparisons to be made between rape cases that included one versus multiple defendants. This has important implications for the prosecution of MPR cases. Despite low conviction rates in rape cases (RAINN, 2025b) as well as concerns over the “she-said-he-said” battle that often characterizes many rape cases, our results suggest that laypeople take cases of MPR seriously. This aligns with Horvath and Gray’s (2013) findings that most MPR cases that proceeded to trial resulted in a conviction as well as longer sentences (compared to cases where perpetrators were charged with lesser crimes). Coupled with the present study, this suggests that prosecutors should pursue cases of MPR. Our results also offer suggestions for how best to do so.

By examining not only verdict decisions but also measures of mock jurors’ perceptions of the victim and defendant(s) in these cases, our results offer novel contributions to the literature and provide insight into how jurors may differentially evaluate acquaintance rape cases that involve multiple defendants. Our results suggest that perceptions of victim helplessness and a lack of defendant credibility and sympathy may be particularly relevant to jurors’ decision making in MPR cases. As such, prosecutors may want to focus on introducing evidence that highlights a victim’s helplessness (e.g., their inability to resist the attack) as well as evidence that implicates the defendants’ credibility, lack of concern for the victim, or lack of appreciation for the wrongfulness of their actions.

Drawing attention to the victim’s helplessness, in particular, may be fruitful given that victims often do not resist their attackers during MPR because they are outnumbered, overpowered, and fear the added violence against them (Ullman, 2013). This can be problematic because jurors may perceive a lack of resistance as indicative of consent (Ullman, 2013). Thus, by highlighting victim helplessness in MPR cases, this may help dispel the myth that a lack of resistance is equivalent to giving consent. In doing so, it may help address the crucial legal issue of establishing the nonconsensual nature of the attack. Indeed, as the serial indirect effects revealed, higher perceptions of victim helplessness were associated with greater defendant blame and an increased likelihood of voting guilty.

Regarding judgments about the victim and defendant, we should note that the lack of other significant findings was perplexing. For example, prior studies investigating legal decision making in rape cases have found an effect of various independent variables on sympathy toward the victim: Golding et al. (2016, Exp. 3) found that, when a rape victim was impeached by the defense attorney, mock jurors had less sympathy for the victim and in turn were less likely to vote guilty compared to when the victim was not impeached. However, this effect was not evident, and it is unclear why mock jurors, who felt that a victim was more helpless in an MPR case than a single-defendant case, would not also have more sympathy toward the victim. Of course, additional research will be necessary to understand the specific perceptions and emotions that mock jurors experience when faced with an MPR case.

Data from the present study also allowed us to better understand the underlying reasons for a guilty verdict, and the cognitive representations of mock jurors who voted guilty. Regarding the former, we examined indirect effects. This analysis revealed the factors that were associated with an increase in guilty verdicts for MPR cases compared to when there was a lone defendant. The use of indirect effect analyses continues a trend in the rape and legal decision-making literature (e.g., Osborn et al., 2021), as well as the literature examining other types of victimization in the courtroom (e.g., child sexual assault, see, e.g., Jones et al., 2020). In the present study, the parallel indirect effects revealed that the MPR (vs. lone perpetrator) case led to lower defendant credibility, lower defendant sympathy, more anger toward the defendant, and higher perceived victim helplessness. These were all associated with a greater likelihood of voting guilty. Of note, by including several measures of perceptions of the victim and defendant, this analysis allowed us to determine which of these perceptions were the strongest predictors of guilty verdicts in MPR cases. Results therefore suggest that, in cases of MPR, prosecutors may want to focus on evidence that implicates the credibility of defendants and, as noted above, emphasizes the wrongfulness of the act as well as the victim’s helplessness.

Our examination of indirect effects also included a serial indirect effect. Specifically, we found that, compared to lone perpetrator cases, in MPR cases, mock jurors perceived the victim to be more helpless, which was associated with them finding the defendants more blameworthy and ultimately being more likely to convict. The serial indirect effect, in the context of MPR, is especially important for two reasons. First, the present study highlights the importance of perceptions of victim helplessness in MPR cases (see Schafran, 2005), a dependent variable typically not assessed in prior published acquaintance rape and legal decision-making research that included a single defendant (for review, see Burke et al., 2025). It is unclear why this measure was not included in prior studies, although it is possible that researchers felt that examining other aspects of a rape victim’s behavior (e.g., ability to consent, ability to communicate) was sufficient. Alternatively, the issue of victim helplessness may be deemed critical only in MPR cases, where a victim’s helplessness may be increased. Second, this finding suggests that victimization and legal decision-making researchers must be open to more complex underlying explanations for certain effects. In the past, researchers have analyzed indirect effects involving single variables (e.g., victim empathy) or parallel indirect effects (Jones et al., 2020; Mecikalski et al., 2024). The present results may imply the importance of greater analytic exploration, such as future research examining serial indirect effects, moderated indirect effects, and even moderated serial indirect effects (see Preacher & Hayes, 2004).

The cognitive representation of those who voted guilty was examined through the construction of cognitive networks. As described earlier, these networks were derived from participants’ reasons for their guilty verdicts. The networks for the three-defendant condition showed that mock jurors were attuned to when a case involved MPR by offering the presence of multiple defendants as a critical reason for their guilty verdicts. This qualitative finding supports the quantitative data described earlier (e.g., more guilty verdicts for the three-defendant versus the one-defendant condition). Moreover, this finding supports the viewpoint of others (Horvath & Kelly, 2009; Woodhams & Horvath, 2013) regarding the unique status of MPR as a type of sexual crime. It is also worth noting that prior work has found that the more witnesses who testify for the defense, the less likely jurors are to convict (Werner et al., 1985). Yet, in cases of MPR, our results suggest that prosecutors may not need to be as concerned with this, especially if they implicate the credibility of the other defendant–perpetrator–witnesses by, for example, noting that their testimony reflects their own self-interests and emphasizing the nonconsensual and overpowering nature of the attack.

In addition, as Horvath and Gray (2013) first advocated for MPR cases in England and Wales, prosecutors and State Attorneys might consider a greater application of “joint criminal enterprise” or “common purpose,” which is a legal doctrine that allows for the charging and prosecution of individuals who were involved in a crime, even if they did not directly participate in it. Currently, Massachusetts is the only state that explicitly refers to this in their sexual assault statutes (Massachusetts General Laws. 265 §22, n.d.; National Crime Victim Law Institute & National Women’s Law Center, 2016); however, broader applicability of this doctrine would ensure that individuals who were complicit in MPR indirectly (e.g., by observing the attack) would also be prosecuted and held to justice.

With respect to the manipulation of victim intoxication, although verdicts did not differ as a function of whether the victim was intoxicated or sober, jurors were sensitive to how intoxication may have affected the victim. This was evident by jurors perceiving the intoxicated (vs. sober) victim to be more helpless, less able to communicate or consent, and more likely to be viewed as vulnerable by the defendant. These findings contrast with prior work showing that mock jurors were more punitive toward intoxicated (vs. sober) victims (e.g., perceiving them to be less credible; Martin & Monds, 2024). Given recent attention to sexual abuse, sexual harassment, and rape in mainstream society (e.g., #MeToo Movement), perhaps participants in the present study were more attuned to issues of sexual violence and more likely to combat negative stereotypes surrounding victims of such violence. This is promising, given that cases of MPR, like other types of rape, often involve the use of alcohol (Richer et al., 2017). Given prior work, it may behoove prosecutors to question jurors during voir dire to gauge jurors’ attitudes and stereotypes regarding the credibility of victims who were under the influence of drugs or alcohol to ensure that the jury is not prejudiced.

Finally, our results add to the robust effect that participant gender has in predicting outcomes in sexual assault cases (e.g., Belyea & Blais, 2023; for review, see Golding et al., 2023), with women rendering more pro-victim judgments (i.e., higher victim credibility, lower victim blame, less anger toward the victim, more anger toward the defendant) than men. These results are consistent with feminist theory. Specifically, feminist scholars argue that sexual violence against women stems from societal norms (e.g., traditional gender roles and socialization) and systems (e.g., patriarchy, the objectification of women in the media) that emphasize male dominance, aggression, and power over women (e.g., Brownmiller, 1975; McPhail, 2016), and in doing so, it promotes a culture of rape (for review, see Canan & Levand, 2019). This may explain why, compared to women, men report a greater acceptance of rape myths and are more likely to blame victims (e.g., Barnett et al., 2018; Fakunmoju et al., 2021). In contrast, according to feminist theory, women are constantly fearful of being victims of rape (Rennison, 2014), which may explain why women (vs. men) mock jurors display more sympathy toward victims of rape and are more likely to render convictions in these cases (Angelone et al., 2007). Judicial instructions directing jurors to focus on the evidence at hand and whether it meets the burden of proof may be beneficial in preventing jurors from relying on stereotypes by encouraging them to focus strictly on the case facts.

### Limitations

Although the present study contributes to the rape and legal decision-making literature, it has limitations. First, there might be a concern about the validity of online studies (e.g., Webb & Tangney, 2022). However, Gosling et al. (2004) found that online results led to similar results as those of traditional (in-person) studies. In addition, we believe that our use of manipulation- and attention-check questions attenuates concerns about the integrity of our results by ensuring that participants were aware of the experimental manipulations and by motivating participants to pay attention. Finally, the present sample included jury-eligible online participants who ranged in age and gender. This point, along with Bornstein et al.’s (2017) meta-analysis of mock juror research that showed very few differences as a function of sample type, offers support for the generalizability of our results.

Second, the present study did not include jury deliberations; only individual juror decisions were assessed. Some (e.g., Diamond, 1997; see also Weiner et al., 2011) have argued that jury deliberations may offer a better grasp of trial information than not having deliberations. Nonetheless, Diamond stated that “…the patterns of responses obtained from studies of individual jurors will generally predict jury outcomes…” (p. 565). The goal of the present experiment was to provide an initial investigation of legal decision making involving multiple perpetrators in a rape trial, a topic for which there is a dearth of research. We acknowledge that it will be critical to investigate this issue using more ecologically valid paradigms.

Third, we focused on cases involving adult men as perpetrators and adult women as victims because adult women are among those at the highest risk of sexual victimization in the US (RAINN, 2025a). Nonetheless, this limits the generalizability of our findings and precludes conclusions from being drawn for cases involving other populations (e.g., men, children).

Finally, our methodology included the use of brief trial summaries. As the first study to experimentally examine MPR and legal decision making, we felt that using such a methodology was essential to ensure a high degree of experimental control and allow us to establish a basic understanding of the causal role that MPR has in the courtroom. In doing so, we believe that the present study lays the groundwork for future research to examine this topic. As with the point raised above, we hope that future researchers will use this initial study as a starting point for further investigations using more ecologically valid materials.

## 5. Conclusions

The present study extended the limited research that exists on MPR and legal decision making (see Horvath & Woodhams, 2013) by demonstrating (in a controlled experiment) that mock jurors are more pro-victim (e.g., render more guilty verdicts, rate a victim as more helpless) when dealing with an MPR case compared to a rape case involving a lone defendant. Given that MPR cases are not unique (da Silva & Woodhams, 2019; RAINN, 2024; Woodhams & Horvath, 2013), this study is an important step in understanding factors that increase the likelihood that victims of MPR will receive justice and that perpetrators face their day in court. Hopefully future research will explore other factors that impact legal decision making with these cases, including MPR cases that involve stranger rape and cases that involve LGBTQ+ victims. As we continue to investigate MPR in the courtroom, we hope that the knowledge gained will lead to a greater understanding of which factors will yield a conviction and may prevent the victimization of others in the future.

## Figures and Tables

**Table 1:** Means (standard deviations) of the dependent variables.

	One Defendant								Three Defendants							
	Intoxicated				Sober				Intoxicated				Sober			
	Men		Women		Men		Women		Men		Women		Men		Women	
	*M*	*SD*	*M*	*SD*	*M*	*SD*	*M*	*SD*	*M*	*SD*	*M*	*SD*	*M*	*SD*	*M*	*SD*
Verdict	0.37	0.49	0.32	0.48	0.37	0.50	0.45	0.51	0.48	0.51	0.76	0.44	0.67	0.48	0.67	0.48
Victim																
Credibility	4.48	1.60	4.66	1.73	4.67	1.71	4.83	1.96	4.42	1.54	5.15	1.25	4.89	1.56	5.67	1.35
Blame	3.02	1.70	3.13	1.90	2.71	1.46	2.53	1.72	3.81	1.32	2.65	1.87	3.29	1.97	2.06	1.69
Communicate	4.53	1.66	4.00	2.08	5.95	1.43	5.80	1.77	4.05	1.60	3.53	1.70	6.14	1.46	5.67	1.58
Consent	3.90	1.97	3.05	2.09	5.68	1.63	5.75	1.97	3.55	1.54	2.71	1.90	5.10	1.95	4.92	2.08
Helplessness	4.57	1.72	5.53	1.31	3.16	1.83	3.35	2.18	5.38	1.47	5.82	1.01	3.86	1.93	4.92	1.82
Sympathy	4.63	2.08	4.89	2.31	4.74	1.82	4.95	1.88	4.62	1.53	5.47	1.97	4.52	2.02	5.46	1.93
Anger	1.97	1.50	1.79	1.36	2.53	1.54	1.90	1.25	3.10	1.79	2.29	1.86	2.24	1.79	1.50	1.44
Intoxication	6.40	1.25	6.79	0.54	1.47	1.26	1.00	0.00	6.57	0.60	6.65	0.70	1.24	1.09	1.08	0.41
Defendant																
Credibility	4.13	1.63	4.28	1.76	3.89	1.44	4.00	1.75	3.65	1.86	2.97	1.56	3.17	1.81	2.60	1.57
Blame	5.13	1.66	4.47	2.04	4.71	1.80	5.10	1.66	4.81	1.82	5.91	1.51	5.06	1.74	5.74	1.23
Force	3.53	1.94	3.21	1.44	3.84	1.92	3.95	2.24	3.19	1.82	4.08	1.95	3.70	1.81	4.53	2.04
Vulnerability	4.33	2.02	4.89	1.82	3.26	1.37	3.90	2.05	4.29	1.91	5.49	1.85	3.37	2.15	3.67	2.05
Sympathy	2.97	1.85	3.26	1.82	3.16	1.68	2.95	1.82	2.76	1.57	1.73	1.41	2.43	1.56	1.99	1.72
Anger	3.57	2.18	4.26	2.31	4.26	1.91	4.05	2.28	3.46	2.32	5.63	1.93	4.84	1.98	4.92	2.26
Intoxication	2.60	2.58	2.42	2.52	1.63	1.30	1.15	0.67	1.14	0.48	1.53	1.17	1.43	1.18	1.17	0.56

**Table 2:** Hierarchical regression results.

	*B*	*SE*	*p*	Wald	*OR*	*Nagelkerke R* ^2^
Verdict						
Step 1						0.01
Gender	−0.35	0.31	0.25	1.33	0.70	
Step 2						0.10
Gender	−0.30	0.32	0.35	0.87	0.74	
Defendant Number	1.05	0.32	<0.001	10.84	2.86	
Victim Intoxication	0.24	0.32	0.46	0.55	1.27	
Victim Ratings						
	***B***	***SE***	***p***	***R* ^2^**	**Δ*R*^2^**	***p***
Credibility						
Step 1				0.02	0.02	0.04
Gender	−0.50	0.25	0.04			
Step 2				0.05	0.03	0.12
Gender	−0.45	0.25	0.07			
Defendant Number	0.35	0.24	0.15			
Victim Intoxication	0.33	0.25	0.18			
Blame						
Step 1				0.03	0.03	0.02
Gender	0.64	0.26	0.02			
Step 2				0.05	0.02	0.18
Gender	0.60	0.27	0.03			
Defendant Number	0.15	0.26	0.58			
Victim Intoxication	−0.49	0.27	0.07			
Communication						
Step 1				0.00	0.00	0.41
Gender	0.24	0.29	0.41			
Step 2				0.24	0.24	<0.001
Gender	0.43	0.25	0.09			
Defendant Number	−0.23	0.25	0.36			
Victim Intoxication	1.85	0.26	<0.001			
Consent						
Step 1				0.00	0.00	0.42
Gender	0.27	0.33	0.42			
Step 2				0.23	0.23	<0.001
Gender	0.45	0.29	0.13			
Defendant Number	−0.54	0.29	0.07			
Victim Intoxication	2.04	0.29	<0.001			
Helplessness						
Step 1				0.02	0.02	0.06
Gender	−0.57	0.29	0.06			
Step 2				0.21	0.19	<0.001
Gender	−0.69	0.26	0.01			
Defendant Number	0.87	0.26	0.001			
Victim Intoxication	−1.50	0.26	<0.001			
Sympathy						
Step 1				0.02	0.02	0.06
Gender	−0.57	0.30	0.06			
Step 2				0.02	0.00	0.80
Gender	−0.56	0.30	0.06			
Defendant Number	0.20	0.30	0.51			
Victim Intoxication	0.01	0.30	0.98			
Anger						
Step 1				0.03	0.03	0.02
Gender	0.57	0.24	0.02			
Step 2				0.04	0.01	0.40
Gender	0.56	0.24	0.02			
Defendant Number	0.27	0.24	0.27			
Victim Intoxication	−0.22	0.25	0.38			
Defendant Ratings						
	***B***	***SE***	***p***	***R* ^2^**	**Δ*R*^2^**	***p***
Credibility				0.01	0.01	0.23
Step 1						
Gender	0.32	0.27	0.23			
Step 2				0.10	0.09	<0.001
Gender	0.23	0.26	0.37			
Defendant Number	−0.95	0.26	<0.001			
Victim Intoxication	−0.33	0.26	0.21			
Blame						
Step 1				0.01	0.01	0.16
Gender	−0.36	0.26	0.16			
Step 2				0.03	0.02	0.21
Gender	−0.34	0.26	0.20			
Defendant Number	0.45	0.26	0.08			
Victim Intoxication	0.04	0.26	0.88			
Force Used						
Step 1				0.01	0.01	0.16
Gender	−0.42	0.29	0.16			
Step 2				0.03	0.02	0.19
Gender	−0.35	0.29	0.23			
Defendant Number	0.20	0.29	0.50			
Victim Intoxication	0.48	0.29	0.10			
Victim’s Vulnerability						
Step 1				0.02	0.02	0.09
Gender	−0.53	0.31	0.09			
Step 2				0.10	0.08	<0.001
Gender	−0.66	0.30	0.03			
Defendant Number	0.08	0.30	0.79			
Victim Intoxication	−1.20	0.30	<0.001			
Sympathy						
Step 1				0.01	0.01	0.18
Gender	0.36	0.27	0.18			
Step 2				0.07	0.06	0.01
Gender	0.32	0.26	0.23			
Defendant Number	−0.81	0.26	<0.001			
Victim Intoxication	−0.03	0.26	0.90			
Anger						
Step 1				0.03	0.03	0.04
Gender	−0.71	0.34	0.04			
Step 2				0.05	0.02	0.09
Gender	−0.65	0.34	0.06			
Defendant Number	0.63	0.34	0.06			
Victim Intoxication	0.31	0.34	0.35			

Note. Gender is coded 0 = woman. Defendant number is coded 0 = one. Victim intoxication is coded 0 = intoxicated.

**Table 3:** Parallel indirect effects from number of defendants to verdict.

Direct Effect	Effect	SE	95% CI ^a^
	−1.20	0.68	−2.53, 0.13
Indirect Effects	Effect	SE	95% CI
Victim Credibility	0.21	0.13	−0.04, 0.47
Defendant Credibility	0.53	0.13	0.27, 0.79
Victim Blame	−0.01	0.11	−0.21, 0.20
Defendant Blame	0.22	0.12	−0.02, 0.46
Victim Sympathy	0.07	0.09	−0.11, 0.26
Defendant Sympathy	0.37	0.12	0.14, 0.60
Victim Anger	−0.04	0.05	−0.14, 0.07
Defendant Anger	0.24	0.11	0.02, 0.46
Victim Helplessness	0.24	0.10	0.05, 0.42
Victim Consent	0.08	0.08	−0.07, 0.23
Total	0.72	0.20	0.33, 1.10

^a^ 95% CI is based on 10,000 bootstrap samples. Note. Defendant number is coded 0 = One. Verdict is coded 0 = Not guilty.

## Data Availability

Study materials are available at the link to the supplementary materials listed above.

## Notes

1 We acknowledge that the terms “victim” or “survivor” may be used to refer to someone who has experienced a sexual assault. We use “victim” in this paper because it is more commonly used in the criminal justice context (i.e., the courtroom), which is the focus of the present study.

2 Complicating matters, crime reporting in some countries does not distinguish between MPR and other forms of sexual assault (World Population Review, 2025), making it difficult to determine differences in the prevalence of MPR across countries.

3 Compared to other techniques, cognitive networks can provide more nuanced data by assessing not only the frequency of emerging themes but also visualizing the relations between these themes. They depict the relations between different concepts that participants mentioned in their open-ended responses. Each network contains nodes (i.e., the primary concepts that participants referred to) that are connected by links. Nodes are linked together based on the degree of relationship (e.g., similarity) between them in the open-ended responses. Commonly cited concepts are more central in the network and have more links to other nodes, and closely related concepts are linked directly to each other.

4 Using this method, researchers develop a detailed and realistic case summary of a trial to present to mock jurors in a vignette or other format (e.g., videorecorded slideshow). To increase ecological validity, the trial summary incorporates legally appropriate charges, realistic witnesses, admissible evidence, and pattern jury instructions. Participants are randomly assigned to different trial conditions that are the same except for the experimental manipulation. The structure of the mock trial summary follows an abbreviated version of the steps that occur in a real trial: It begins with opening statements by the prosecution and defense; followed by the prosecution’s presentation of their case, including prosecution witness testimony and defense cross-examination; the defense’s presentation of their case, including defense witness testimony and prosecution cross-examination; closing arguments from the prosecution and defense; and pattern jury instructions. Following this, jurors render a verdict and a series of other case judgments.

5 Because keywords are rank-ordered by their average TF-IDF scores, there is no fixed cutoff score or frequency required for inclusion. Terms with lower scores naturally fall to the bottom of the list and are unlikely to be selected. However, in cases where overall TF-IDF scores or term frequencies are extremely low and tightly clustered (e.g., when the most frequent terms appear only twice across all responses), there is little meaningful variance among terms. This results in distance matrices with limited range, producing networks that are either fully or nearly fully connected. Such networks offer little interpretive value, as they obscure meaningful distinctions between concepts. In these cases, we abandon network analysis in favor of alternative approaches better suited to sparse highly heterogeneous response data.

6 We acknowledge the issue of temporal precedence involved in our indirect-effect analyses. However, we believe that this is justified for several reasons: (a) social psychological research practices do not require temporal precedence in research design to use indirect-effect analyses (Rucker et al., 2011) so long as causality is not being established (Winer et al., 2016). We should note that we do not intend to claim causal effects on the verdict. Moreover, it was necessary to measure perceptions of the victim and defendant after the verdict for methodological reasons given that the verdict was the most important, ecologically relevant outcome measure, and we did not want other measures to influence the ways in which jurors rendered their verdict.

## Supplementary Materials

The following supporting information can be downloaded at: https://osf.io/w2874/?view_only=cd815a674aa04324a96027def93b032e (accessed on 18 June 2025).

## References

[B1-behavsci-15-00844] American Psychological Association (2017). Ethical principles of psychologists and code of conduct (2002, amended effective 1 June 2010, and 1 January 2017).

[B2-behavsci-15-00844] Angelone D. J., Mitchell D., Pilafova A. (2007). Club drug use and intentionality in perceptions of rape victims. Sex Roles.

[B3-behavsci-15-00844] Arya D. (2022). Nirbhaya 10 years on: The lives the Delhi gang rape changed. BBC News.

[B4-behavsci-15-00844] Barnett M., Sligar K. B., Wang C. D. C. (2018). Religious affiliation, religiosity, gender, and rape myth acceptance: Feminist theory and rape culture. Journal of Interpersonal Violence.

[B5-behavsci-15-00844] Basile K. C., Smith S. G., Kresnow M., Khatiwada S., Leemis R. W. (2022). The national intimate partner and sexual violence survey (NISVS): 2016/2017 report on sexual violence.

[B6-behavsci-15-00844] Belyea L., Blais J. (2023). Effect of pretrial publicity via social media, mock juror sex, and rape myth acceptance on juror decisions in a mock sexual assault trial. Psychology, Crime & Law.

[B7-behavsci-15-00844] Bornstein B. H., Golding J. M., Neuschatz J., Kimbrough C., Reed K., Magyarics C., Luecht K. (2017). Mock juror sampling issues in jury simulation research: A meta-analysis. Law and Human Behavior.

[B8-behavsci-15-00844] Bray R. M., Kerr N. L. (1979). Use of the simulation method in the study of jury behavior: Some methodological considerations. Law and Human Behavior.

[B9-behavsci-15-00844] Brownmiller S. (1975). Against our will: Men, women and rape.

[B10-behavsci-15-00844] Buchanan E. M., Scofield J. E. (2018). Methods to detect low quality data and its implication for psychological research. Behavior Research Methods.

[B11-behavsci-15-00844] Bulus M. (2023). pwrss: Statistical power and sample size calculation tools *(R package version 0.3.1)*.

[B12-behavsci-15-00844] Burke K. C., Golding J. M., Neuschatz J. S. (2025). Sexual victimization and legal decision making. Ohio State Journal of Criminal Law.

[B13-behavsci-15-00844] Canan S. N., Levand M. A., O’Donohue W. T., Schewe P. A. (2019). A feminist perspective on sexual assault. Handbook of sexual assault and sexual assault prevention.

[B14-behavsci-15-00844] Crivatu I. M., Horvath M. A., Massey K. (2023). The impacts of working with victims of sexual violence: A rapid evidence assessment. Trauma, Violence, & Abuse.

[B15-behavsci-15-00844] da Silva T., Woodhams J. (2019). Introduction to the special issue on multiple perpetrator sexual offending. Journal of Sexual Aggression.

[B16-behavsci-15-00844] Diamond S. S. (1997). Illuminations and shadows from jury simulations. Law and Human Behavior.

[B17-behavsci-15-00844] Fakunmoju S. B., Abrefa-Gyan T., Maphosa N., Gutura P. (2021). Rape myth acceptance: Gender and cross-national comparisons across the United States, South Africa, Ghana, and Nigeria. Sexuality & Culture.

[B18-behavsci-15-00844] Fansher A. K., Welsh B. (2023). A decade of decision making: Prosecutorial decision making in sexual assault cases. Social Sciences.

[B19-behavsci-15-00844] FBI (2013). Uniform crime report: Crime in the United States.

[B20-behavsci-15-00844] Figueroa T., Winkley L., Wilkens J. (2022). As D.A. considers SDSU gang rape claims, experts explain why such cases are challenging. Los Angeles Times.

[B22-behavsci-15-00844] Golding J. M., Lynch K. R., Renzetti C. M., Pals A. M., Bornstein B. H., Miller M. K. (2023). Beyond the stranger in the woods: Investigating the complexity of adult rape cases in the courtroom. Advances in psychology and law.

[B21-behavsci-15-00844] Golding J. M., Lynch K. R., Wasarhaley N. E. (2016). Impeaching rape victims in criminal court: Does concurrent civil action hurt justice?. Journal of Interpersonal Violence.

[B23-behavsci-15-00844] Golding J. M., Malik S., Jones T. M., Burke K. C., Bottoms B. L., Pozzulo J., Pica E., Sheahan C. (2020). Perceptions of child sexual abuse victims: A review of psychological research with implications for law. Memory and sexual misconduct: Psychological research for criminal justice.

[B24-behavsci-15-00844] Gosling S. D., Vazire S., Srivastava S., John O. P. (2004). Should we trust web-based studies? A comparative analysis of six preconceptions about internet questionnaires. American Psychologist.

[B25-behavsci-15-00844] Hanson R. K., Lee S. C., Thornton D. (2022). Long-term recidivism rates among individuals at high risk to sexually reoffend. Sexual Abuse.

[B26-behavsci-15-00844] Hauser D. J., Moss A. J., Rosenzweig C., Jaffe S. N., Robinson J., Litman L. (2023). Evaluating cloudresearch’s approved group as a solution for problematic data quality on MTurk. Behavior Research Methods.

[B27-behavsci-15-00844] Horvath M., Gray J. M., Horvath M., Woodhams J. (2013). Multiple perpetrator rape in the courtroom. Handbook on the study of multiple perpetrator rape: A multidisciplinary response to an international problem.

[B28-behavsci-15-00844] Horvath M., Kelly L. (2009). Multiple perpetrator rape: Naming an offence and initial research findings. Journal of Sexual Aggression.

[B29-behavsci-15-00844] Horvath M., Woodhams J. (2013). Handbook on the study of multiple perpetrator rape: A multidisciplinary response to an international problem.

[B30-behavsci-15-00844] Illinois Criminal Code. 720 ILCS §§ 5/11-1.20 https://www.ilga.gov/legislation/ilcs/ilcs4.asp?ActID=1876&ChapterID=53&SeqStart=14900000&SeqEnd=16400000.

[B31-behavsci-15-00844] Jenkins G., Schuller R. A. (2007). The impact of negative forensic evidence on mock jurors’ perceptions of a trial of drug-facilitated sexual assault. Law and Human Behavior.

[B32-behavsci-15-00844] Jewkes R., Fulu E., Roselli T., Garcia-Moreno C. (2013). Prevalence of and factors associated with non-partner rape perpetration: Findings from the UN multi-country cross-sectional study on men and violence in asia and the pacific. The Lancet Global Health.

[B33-behavsci-15-00844] Jewkes R., Sikweyiya Y., Dunkle K., Morrell R. (2015). Relationship between single and multiple perpetrator rape perpetration in South Africa: A comparison of risk factors in a population-based sample. BMC Public Health.

[B34-behavsci-15-00844] Johnson N. L., Johnson D. M. (2021). An empirical exploration into the measurement of rape culture. Journal of Interpersonal Violence.

[B35-behavsci-15-00844] Jones T. M., Bottoms B. L., Stevenson M. C. (2020). Child victim empathy mediates the influence of jurors’ sexual abuse experiences on child sexual abuse case judgments: Meta-analyses. Psychology, Public Policy, and Law.

[B36-behavsci-15-00844] Kelly L., Horvath M., Woodhams J. (2013). Naming and defining multiple perpetrator rape: The relationship between concepts and research. Handbook on the study of multiple perpetrator rape: A multidisciplinary response to an international problem.

[B37-behavsci-15-00844] Kentucky Penal Code. §§ 510.040-510.060 https://apps.legislature.ky.gov/law/statutes/statute.aspx?id=19761.

[B38-behavsci-15-00844] Lees S. (2002). Carnal knowledge: Rape on trial.

[B39-behavsci-15-00844] Levi M. M., Lynch K. R., Golding J. M. (2022). Strength versus sensitivity: The impact of attorney gender on juror perceptions and trial outcomes in a rape case. Violence Against Women.

[B40-behavsci-15-00844] Lynch K. R., Wasarhaley N. E., Golding J. M., Simcic T. A. (2013). Who bought the drinks? Juror perceptions of intoxication in a rape trial. Journal of Interpersonal Violence.

[B41-behavsci-15-00844] Martin E., Monds L. A. (2024). The effect of victim intoxication and crime type on mock jury decision-making. Psychology, Crime & Law.

[B42-behavsci-15-00844] Massachusetts General Laws. 265 §22 https://malegislature.gov/Laws/GeneralLaws.

[B43-behavsci-15-00844] MATLAB (2024). MATLAB *[version 24.1.0.2578822 (R2024a)]*.

[B44-behavsci-15-00844] McPhail B. A. (2016). Feminist Framework Plus: Knitting feminist theories of rape etiology into a comprehensive model. Trauma, Violence, & Abuse.

[B45-behavsci-15-00844] Mecikalski A. A., Golding J. M., Burke K. C., Neuschatz J. S. (2024). Legal decision-making in an adult rape case involving DNA evidence. Violence Against Women.

[B46-behavsci-15-00844] National Crime Victim Law Institute, National Women’s Law Center (2016). Sexual assault statutes in the United States chart.

[B47-behavsci-15-00844] O’Callaghan E., Lorenz K., Ullman S. E., Kirkner A. (2021). A dyadic study of impacts of sexual assault disclosure on survivors’ informal support relationships. Journal of Interpersonal Violence.

[B48-behavsci-15-00844] Osborn K., Davis J. P., Button S., Foster J. (2021). Juror decision making in acquaintance and marital rape: The influence of clothing, alcohol, and preexisting stereotypical attitudes. Journal of Interpersonal Violence.

[B49-behavsci-15-00844] Peterson C., DeGue S., Florence C., Lokey C. N. (2017). Lifetime economic burden of rape among US adults. American Journal of Preventive Medicine.

[B50-behavsci-15-00844] Preacher K. J., Hayes A. F. (2004). SPSS and SAS procedures for estimating indirect effects in simple mediation models. Behavior Research Methods.

[B51-behavsci-15-00844] Przybylski R. (2015). Recidivism of adult sexual offenders.

[B53-behavsci-15-00844] RAINN (2024). Perpetrators of sexual violence: Statistics.

[B54-behavsci-15-00844] RAINN (2025a). Victims of sexual violence: Statistics.

[B55-behavsci-15-00844] RAINN (2025b). What to expect from the criminal justice system.

[B56-behavsci-15-00844] Rape Prevention and Education Program (2024). https://www.cdc.gov/sexual-violence/programs/.

[B52-behavsci-15-00844] R Core Team (2024). R: A language and environment for statistical computing.

[B57-behavsci-15-00844] Regan P. C., Baker S. J. (1998). The impact of child witness demeanor on perceived credibility and trial outcome in sexual abuse cases. Journal of Family Violence.

[B58-behavsci-15-00844] Rennison C. M., Bruinsma G., Weisburd D. (2014). Feminist theory in the context of sexual violence. Encyclopedia of criminology and criminal justice.

[B59-behavsci-15-00844] Richer L. A., Fields L., Bell S., Heppner J., Dodge J., Boccellari A., Shumway M. (2017). Characterizing drug-facilitated sexual assault subtypes and treatment engagement of victims at a hospital-based rape treatment center. Journal of Interpersonal Violence.

[B60-behavsci-15-00844] Ross L. (2024). Mock juries, real trials: How to solve (some) problems with jury science. Journal of Law and Society.

[B61-behavsci-15-00844] Rosseel Y. (2012). lavaan: An R package for structural equation modeling. Journal of Statistical Software.

[B62-behavsci-15-00844] Rucker D. D., Preacher K. J., Tormala Z. L., Petty R. E. (2011). Mediation analysis in social psychology: Current practices and new recommendations. Social and Personality Psychology Compass.

[B63-behavsci-15-00844] Ruva C. L., Smith K. D., Sykes E. C. (2023). Gender, generations, and guilt: Defendant gender and age affect jurors’ decisions and perceptions in an intimate partner homicide trial. Journal of Interpersonal Violence.

[B64-behavsci-15-00844] Saulnier A., Burke K. C., Bottoms B. L. (2019). The effects of body-worn camera footage and eyewitness race on jurors’ perceptions of police use of force. Behavioral Sciences & the Law.

[B65-behavsci-15-00844] Schafran L. H. (2005). Barriers to credibility: Understanding and countering rape myths.

[B67-behavsci-15-00844] Schuller R. A., Ryan A., Krauss D., Jenkins G. (2013). Mock juror sensitivity to forensic evidence in drug facilitated sexual assaults. International Journal of Law and Psychiatry.

[B66-behavsci-15-00844] Schuller R. A., Wall A. M. (1998). The effects of defendant and complainant intoxication on mock jurors’ judgements of sexual assault. Psychology of Women Quarterly.

[B68-behavsci-15-00844] Schvaneveldt R. W. (1990). Pathfinder associative networks: Studies in knowledge organization.

[B69-behavsci-15-00844] Spohn C., Tellis K. (2019). Sexual assault case outcomes: Disentangling the overlapping decisions of police and prosecutors. Justice Quarterly.

[B70-behavsci-15-00844] Statista (2022). Number of reported forcible rape cases in the United States from 1990 to 2022.

[B71-behavsci-15-00844] Stuart S. M., McKimmie B. M., Masser B. M. (2019). Rape perpetrators on trial: The effect of sexual assault–related schemas on attributions of blame. Journal of Interpersonal Violence.

[B72-behavsci-15-00844] Tenney E. R., MacCoun R. J., Spellman B. A., Hastie R. (2007). Calibration trumps confidence as a basis for witness credibility. Psychological Science.

[B73-behavsci-15-00844] Ullman S. E., Horvath M., Woodhams J. (2013). Multiple perpetrator rape victimization: How it differs and why it matters. Handbook on the study of multiple perpetrator rape: A multidisciplinary response to an international problem.

[B74-behavsci-15-00844] Vetten L., Haffejee S. (2005). Gang rape: A study in inner-city Johannesburg. South African Crime Quarterly.

[B75-behavsci-15-00844] Walinchus L., Smyser L., Murphy J. (2025). A vanishingly small number of violent sex crimes end in conviction, NBC News investigation shows. NBC News.

[B76-behavsci-15-00844] Wall A. M., Schuller R. A. (2000). Sexual assault and defendant/victim intoxication: Jurors’ perceptions of guilt. Journal of Applied Social Psychology.

[B77-behavsci-15-00844] Webb M. A., Tangney J. P. (2022). Too good to be true: Bots and bad data from Mechanical Turk. Perspectives on Psychological Science.

[B78-behavsci-15-00844] Weiner R. L., Krauss D. A., Lieberman J. D. (2011). Mock jury research: Where do we go from here?. Behavioral Sciences and the Law.

[B79-behavsci-15-00844] Werner C. M., Strube M. J., Cole A. M., Kagehiro D. K. (1985). The impact of case characteristics and prior jury experience on jury verdicts. Journal of Applied Social Psychology.

[B80-behavsci-15-00844] Wieberneit M., Thal S., Clare J., Notebaert L., Tubex H. (2024). Silenced survivors: A systematic review of the barriers to reporting, investigating, prosecuting, and sentencing of adult female rape and sexual assault. Trauma, Violence, & Abuse.

[B81-behavsci-15-00844] Wiley T. R., Bottoms B. L. (2013). Attitudinal and individual differences influence perceptions of mock child sexual assault cases involving gay defendants. Journal of Homosexuality.

[B82-behavsci-15-00844] Winer E. S., Cervone D., Bryant J., McKinney C., Liu R. T., Nadorff M. R. (2016). Distinguishing mediational models and analyses in clinical psychology: Atemporal associations do not imply causation: Temporal and atemporal mediation. Journal of Clinical Psychology.

[B83-behavsci-15-00844] Woodhams J., Horvath M., Horvath M., Woodhams J. (2013). Introduction. Handbook on the study of multiple perpetrator rape: A multidisciplinary response to an international problem.

[B84-behavsci-15-00844] World Population Review (2025). Rape statistics by country.

